# Utilization of Classification Learning Algorithms for Upper-Body Non-Cyclic Motion Prediction

**DOI:** 10.3390/s25051297

**Published:** 2025-02-20

**Authors:** Bon H. Koo, Ho Chit Siu, Dava J. Newman, Ellen T. Roche, Lonnie G. Petersen

**Affiliations:** 1 Department of Mechanical Engineering, Massachusetts Institute of Technology, Cambridge, MA 02139, USA; bkoo1104@mit.edu (B.H.K.);; 2 Lincoln Laboratory, Massachusetts Institute of Technology, Lexington, MA 02421, USA; hoseasiu@mit.edu; 3 Department of Aeronautics and Astronautics, Massachusetts Institute of Technology, Cambridge, MA 02139, USA; 4 Institute for Medical Engineering and Science, Massachusetts Institute of Technology, Cambridge, MA 02139, USA

**Keywords:** classification, deep learning neural network, KNN, motion prediction, sEMG, neural control of motion

## Abstract

This study explores two methods of predicting non-cyclic upper-body motions using classification algorithms. Exoskeletons currently face challenges with low fluency, hypothesized to be in part caused by the lag in active control innate in many leader–follower paradigms seen in today’s systems, leading to energetic inefficiencies and discomfort. To address this, we employ k-nearest neighbor (KNN) and deep learning models to predict motion characteristics, such as magnitude and category, from surface electromyography (sEMG) signals. Data were collected from six muscles located around the elbow. The sEMG signals were processed to identify significant activation changes. Two classification approaches were utilized: a KNN algorithm that categorizes motion based on the slopes of processed sEMG signals at change points and a deep neural network employing continuous categorization. Both methods demonstrated the capability to predict future voluntary non-cyclic motions up to and beyond commonly acknowledged electromechanical delay times, with the deep learning model able to predict, with certainty at or beyond 90%, motion characteristics even prior to myoelectric activation of the muscles involved. Our findings indicate that these classification algorithms can be used to predict upper-body non-cyclic motions to potentially increase machine interfacing fluency. Further exploration into regression-based prediction models could enhance the precision of these predictions, and further work could explore their effects on fluency when utilized in a tandem or wearable robotic application.

## 1. Introduction

State-of-the-art human–robot tandem applications, such as exoskeletons, are often ineffective due to factors related to seamlessness, energetics, and subjective experience. Among the shortcomings in actuation (motor design, bandwidth, backdrivability, and more) and interfacing (mounting point imprecision, repeatability in interfacing, mounting stiffness, and more), the issue of low fluency [1] stands as one of the most fundamental. While other issues related to exoskeleton design are actively being addressed, with many notable breakthroughs in recent years, the issue of fluency remains relatively stagnant [2]. Fluency, in this context, refers to the perceived and objective seamlessness between a human operator and a robotic agent.

Low fluency is often considered one of the primary reasons behind the inefficiency, subjective discomfort, and increased injury risk in modern human–robot tandem systems. The exact causes of low fluency in exoskeleton applications, in particular, are not yet universally agreed upon. However, the control paradigm implemented in these applications is believed to play a significant role in creating low-fluency interactions. A common and popular model of leader–follower exoskeleton control [3] requires the actuators to lag behind the operator’s motion, which is often hypothesized as the cause of low fluency. Therefore, it is possible—if not likely—that higher fluency could be achieved by eliminating the leader–follower lag. One approach, explored in prior work, including studies discussed in the existing literature, is to anticipate the human agent’s movements through prediction, thus enabling true simultaneous actuation in theory.

A number of studies have shown that various classification paradigms can perform well in motion prediction applications. While most studies aligned with this one involve the use of raw physiological data, such as sEMG input, as features for classification [4], more macroscopic features, such as motion trajectories [5], motion capture [6], and video [7], can also be used for prediction classification. However, the outputs of previous studies utilizing these classification algorithms are limited to identifying motion states or activation states. To improve fluency, a higher-resolution prediction may be required.

The aim of this experiment is to demonstrate the ability of classification algorithms, both k-nearest neighbor (KNN) and deep learning-based, to predict a more granular set of motions using only electromyography (EMG). By leveraging the benefits of KNN classification and, alternatively, using a more complex deep learning algorithm, it is possible to identify additional features, such as the magnitude and type of motion, and predict future voluntary non-cyclic activities before the physical start of motion. This study shows the use of these algorithms to predict the magnitude and type of physical motion in advance of movement. These results suggest that continuous motion prediction (i.e., regression) should be possible using similar techniques.

## 2. Background

### 2.1. Electromechanical Delay (EMD)

It is classically understood that there exists a time lag between the electrostimulation of the primary motor end plate of a muscle and its mechanical motion [8,9,10]. The electrical activation of a muscle leads its physical motion by a variable yet detectable period of time. This time difference is known to vary based on several parameters, including the intended angular velocity, the mass of the target body part, individual physiological differences, and more. The cause of this electromechanical delay (EMD) is not universally agreed upon. There are kinematic causes, such as the elasticity and damping factors of connecting tissues, in addition to electromechanical causes, such as the time elapsed in the cascading activation of the entire muscle group’s fibers. Regardless, most studies that note the existence of EMD agree that physical motion is only detectable some time after activity at the motor plates of the muscles involved. This delay, depending on the source, is shown to be on the order of 50 to 200 ms, depending on the impending motion type and magnitude, among other variables.

### 2.2. Fluency

The measure of seamlessness between a human and a robotic agent(s) working together can be defined as fluency. The method of fluency measurement depends on the application but generally incorporates subjective and objective metrics (such as those defined by Hoffman et al. [1]), correlating measured data with qualitative surveys or behavioral observations. Many human–machine tandem applications, such as wearables or exoskeletons, exhibit low fluency in contemporary implementations. This low fluency is often cited as contributing to higher injury risk, lower metabolic energy efficiency, and reduced user comfort and effectiveness. Several factors are acknowledged as contributing to low fluency, particularly in the exoskeleton context. A primary factor is the interfacing problem, in which a wearable robot’s actuation does not align with that of the user, leading to perceived and measurable reductions in performance and effectiveness [11].

### 2.3. Classification

In this study, two broadly different classification algorithm architectures are used.

#### 2.3.1. KNN

K-nearest neighbor (KNN) classification is a method through which data points are classified according to the (potentially weighted) votes of their k-nearest labeled data points. While KNN classification is not designed for continuous applications, the task of continuous motion prediction can be discretized for KNN use by categorizing motion predictions into labels.

#### 2.3.2. Deep Neural Network Classification

This study also demonstrates predictive classification capabilities using a deep neural network (DNN). While KNN is often considered a “lazy learning” algorithm due to its lack of a training phase, instance-based paradigm, and simple categorization criterion based solely on a distance metric, DNNs offer much greater intricacy compared to KNN-based classification, albeit through a black-box process. One advantage of DNN motion prediction algorithms is their constant-time operation in which processing resources do not scale with the number of labeled examples. If done correctly, this allows them to categorize subsequent data points with performance similar to previous classifications, which is advantageous for motion prediction purposes [12]. While deep learning neural networks have been investigated for motion prediction [13], and sometimes even in conjunction with surface electromyography (sEMG) to predict one-degree-of-freedom knee angles during gait [14], their use in non-cyclic, multi-degree-of-freedom upper-body prediction has not yet appeared in the literature.

### 2.4. Predicting Motion

It stands to reason that if the leader–follower robotic control paradigm—where the robotic agent detects the user’s motion and attempts to keep up to the best of the controller’s ability—is a cause of low fluency (as mentioned in Section 1), then one way to improve these fluency metrics is to actuate synchronously with the user, which requires prediction. Indeed, predicting motion using muscle activation information has shown promise in small, well-defined motion applications, such as hand gripping and releasing [11]. However, prediction in compound, gross-limb motion has yet to be conclusively demonstrated. In this study, we present results from KNN and DNN classification algorithms trained to predict gross-limb, multi-DoF (multi-degree-of-freedom) arm motions and assert that larger, more complex motions can also be predicted using various machine learning methods. We further suggest that utilizing this prediction (in whichever form is most appropriate and highest performing) could be a method to increase fluency in human–robot tandem applications.

### 2.5. Guiding Assumptions

The first assumption made in this study is that the motion of the arm can be identified by observing the activity of a select group of muscles. The arm alone contains 27 known muscles; however, studying every single muscle group would introduce numerous compounding measurement errors, including but not limited to crosstalk error, environmental electric noise, probe placement variation, and overfitting of the classification model. Instead, six of the largest muscles were selected for this study. It is reasonable to conclude that if classification and prediction work with these 6 sample muscles, they would also work with all 27, provided measures are implemented to counteract potential error sources. The primary difference between this experiment and a hypothetical one involving all known muscle groups lies in the size of the dataset, assuming that unaccounted-for error sources are negligible in the experimental environment presented in this study. It is still important to incorporate an understanding of the underlying physiological model when selecting the subset of muscles studied in a particular study. For this, muscles should be selected based on known activation patterns or activation theories, such as the theory of modular recruitment [15], which may guide muscle selection in a model guided by physiological understanding.

The second assumption is that the findings of Schurger et al. and Siu et al., along with other sources investigating EMD and readiness potential mentioned in Section 2.1, are applicable and consistent with the motions explored in this study [4,16]. Due to limitations in measurement devices, this study does not establish a ground-truth correlation between the initiation of the electrical impulse signaling muscle motion and the resulting physiological movement. Instead, it is assumed that physiological motion begins between 50 ms and 190 ms after the initiation of myoelectric activity in the corresponding muscle groups, in accordance with prior work on EMD. As such, any categorization or identification of motion and its qualities occurring before this 50 ms to 190 ms window is considered a prediction of motion in this study, as exemplified by Huang et al. [14].

## 3. Methods

This study takes two distinct approaches to demonstrating the use of prediction algorithms to generate classifications of sEMG-based motion information. While the data were shared, each method features a distinct processing and interpretation method, which are discussed below.

### 3.1. Data Collection

All data used in this study were collected using the same setup. Any distinct motions of the arm were assumed to be fully described by six muscles: Brachioradialis, Biceps Brachii, Triceps Brachii, Deltoid Posterior, Deltoid Medius, and Deltoid Anterior. Surface electromyography (sEMG) probes were placed on each of the listed muscles for a total of six channels. EMG data were collected using the Delsys (Natick, MA, USA) Bagnoli system and an accompanying National Instruments (Austin, TX, USA) DAQ. Each probe was placed approximately at the center of the respective muscle group, where the motor neuron enters the muscle and where the electrical signal strength is strongest. The placement of these probes, while supervised, was performed by the subjects following a series of visual and photographic instructions. The probe placement was reviewed by experimenters for validity, alignment, and consistency, and subjects were instructed to redo it if necessary. The skin at the probe application sites was cleaned using isopropyl alcohol to ensure proper adhesion and signal quality. All six probes shared a common ground, which was attached to a bony surface on the elbow. The experimental space, although not specifically designed to mitigate EMF interference, was selected based on a prior survey to identify an area with the lowest noise floor. This was determined by conducting dry runs with unattached probes and comparing data from different areas of the space based on the standard deviation magnitude of the signal noise.

**Figure A1:**
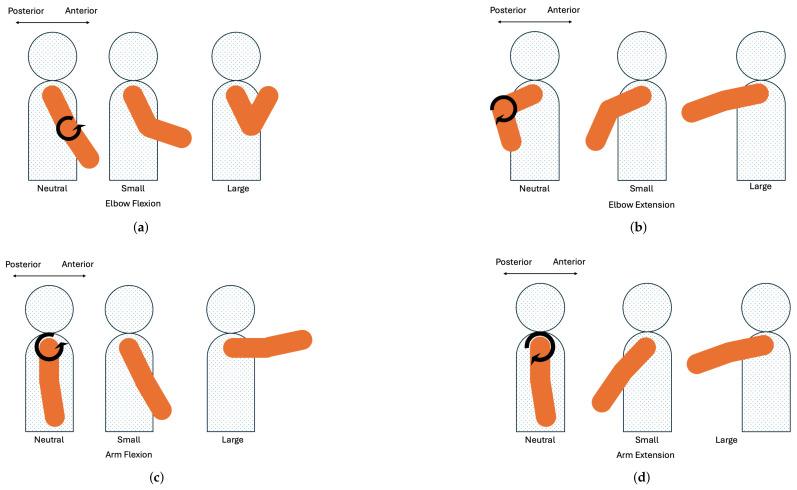

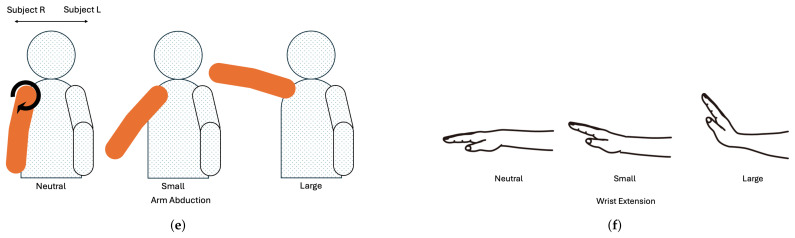
A series of diagrams graphically representing the different motions prescribed to subjects. The exact magnitude (such as small or large) was up to the interpretation of the subject based on a visual guide without strict enforcement. Subfigure (**a**) is a visual representation of a subject performing the small or large elbow flexion motion from a lateral view, with torque application denoted with an arrow. Subfigure (**b**) is a visual representation of a subject performing the small or large elbow extension motion from a lateral view, with torque application denoted with an arrow. Subfigure (**c**) is a visual representation of a subject performing the small or large arm flexion motion from a lateral view, with torque application denoted with an arrow. Subfigure (**d**) is a visual representation of a subject performing the small or large arm extension motion from a lateral view, with torque application denoted with an arrow. Subfigure (**e**) is a visual representation of a subject performing the small or large arm abduction motion from a frontal view, with torque application denoted with an arrow. Subfigure (**f**) is a visual representation of a subject’s lower arm and wrist performing the small or large wrist extension motion from a lateral view.

A total of six types of motions were performed using the subject’s dominant arm, with each motion repeated at two different magnitudes (subjectively determined but not enforced), resulting in 12 motion categories: wrist extension, elbow flexion, elbow extension, supinated arm flexion, pronated arm abduction, and neutral arm extension. Each of the 12 motions was repeated 25 times (organized into five rounds of five trials each), yielding a total of 300 individual motion trials per subject. Each trial consisted of data collected from six channels of EMG signals corresponding to the observed muscles. All channels were sampled at a rate of 1 kHz for the duration required to complete each round of five trials. The motions prescribed are visually illustrated in Figure A1.

This data collection was conducted with six subjects. Subjects underwent a screening process to ensure they met specific criteria, including the absence of physical impairments affecting the upper body or extremities. The final subject pool consisted of four male and two female participants aged 20 to 26, without upper-body impairments. Recruitment was blind to fitness level, and the subjects were not provided with preparatory guidance before data collection. This approach aimed to capture a broad range of natural human motion data and ensure that the resulting prediction algorithm would not be biased by factors such as muscle mass, EMG amplitude, hydration level, or other individual differences.

### 3.2. KNN-Based Classification Algorithm

The raw EMG data collected were pre-processed and conditioned through the following means: A Butterworth filter (4th-order bandstop filter, cutoff frequencies at 58 and 61 Hz, experimentally determined for the experiment environment) was applied in order to exclude the 60-cycle electrical noise present in the environment. Then, the data were centered by subtracting the mean value of each trial from every data point of the corresponding trial, creating a zero-mean signal. Further, a 4th-order Butterworth bandpass filter with cutoff frequencies of 25 and 250 Hz (determined experimentally given the input data, with filter cutoff frequencies modulated based on manufacturer guidance for filter design) was applied. Using these filters, an envelope for the raw data was created. While the values of these filters were determined based on the experimental setup, equipment used, and environmental noise, the methodology was closely incorporated from prior work that also features filtering EMG signals with the intent to extract features. In order to counteract the non-linear phase effects of applying Butterworth filters, a zero-phase filtering approach was used where the filter is applied both forward and backward to the signal.

The envelope signal was then further processed to identify “change points”. In this study, a change point is defined as the region of data where there are the most significant changes in the local mean. In particular, the determination of change points was made using the following logical process:

1. Randomly select *n* points, referred to as candidate change points, and divide the dataset into n+1 sections about these candidate change points.
2. Compute the RMS of the windows immediately before and after each of the *n* candidate change points.
3. Calculate a cost, defined as the inverse of the difference in RMS of the two windows on either side of each candidate change point, thus incentivizing the maximization of the RMS difference flanking each change point.
4. Globally minimize the cost across the entire dataset by observing all possible candidate change point permutations through sweeping a range of *n* (range is selected as a window around the known number of iterations per motion group), thus arriving at *n* change points about which the trajectories change the most drastically.

**Figure 1:**
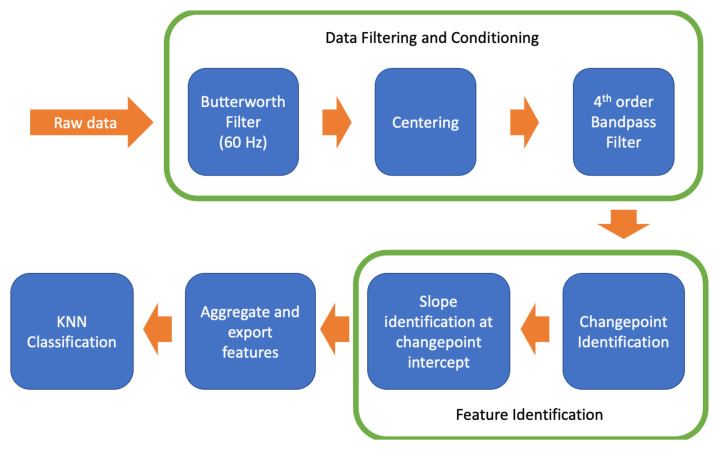
A block diagram of the KNN raw data processing pipeline. The raw data consisted of 6 channels of 5 trials.

It is assumed that a change point in the envelope data indicates a significant change in activation. For this experiment, a single indicator, or feature, was selected for each motion group to facilitate KNN classification: the slope of the processed envelope waveform at these change points. The final dataset consisted of a matrix containing the slope values at every change point for all six tested muscles during each trial, labeled with their corresponding ground-truth motions. Because the data range for each dataset was chosen to encompass a window containing the expected number of iterations per set, the change points, in most cases, aligned with the start and end of each activation cycle. This behavior was consistent with expectations. This matrix was subsequently used as input to a KNN categorization algorithm. A visual representation of this process is shown in Figure 1.

### 3.3. Deep Learning-Based Classification Algorithm

**Figure 2:**
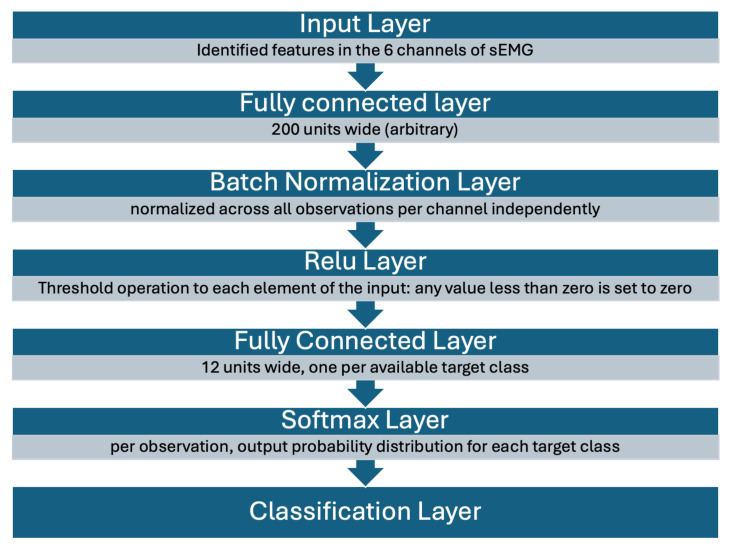
A block diagram of the classification neural network’s structure. The exact design of each layer depends on input parameters, namely the number of features.

The same dataset described in Section 3.1 was utilized to train a neural network. The raw input data consisted of time-series signals from six channels sampled at 1 kHz and labeled with ground-truth motion categories. These data were segmented into rolling windows of a predetermined length. For instance, a window size of 150 samples corresponded to 150 ms of time-series data per window at a sampling rate of 1 kHz. Consequently, each trial was processed into approximately 50,000 to 100,000 rolling windows, depending on the window size. For each window, features were extracted by calculating a single linear least-squares best-fit coefficient, which served as the input to the neural network. The network itself consisted of seven layers, beginning with an input layer and concluding with a classification layer. A diagram of the neural network structure is shown in Figure 2.

### 3.4. Computing Hardware

Most of the training and all inferences presented in this study were conducted using the same local hardware/software combination. The hardware included an Intel i9-10900 CPU, an NVIDIA RTX 2080 Super 8GB GPU, and 16GB of RAM. The software environment included MATLAB 2022b running on the Windows 11 Pro Operating System. Some training, particularly for the DNN portion of this study, was conducted on a high-performance computing unit at MIT SuperCloud, Lincoln Laboratory Supercomputing Center (Lexington, MA, USA).

## 4. Results

### 4.1. KNN Classification Results

**Figure 3:**
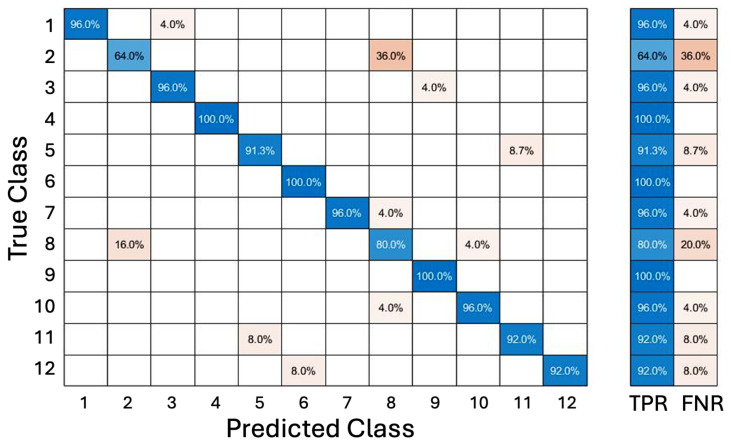
A confusion matrix showing the performance of a Bayesian process-optimized KNN classification prediction algorithm. The class numbers on both axes represent unique motion groups, with 12 different groups in total, as elaborated in Table 2. Overall accuracy approaches 0.93, but certain motion groups are more frequently confused with others, as seen in the off-diagonals. Also shown are the true positive rate (TPR) and the false negative rate (FNR).

The KNN algorithm demonstrated relatively high accuracy in categorizing the different motion groups, with a sample size of 25 trials per motion group per subject. For validation, 10% of the experimental data (rounded to the nearest integer) was used to evaluate the model. Each change point, as described in Section 3.2, was detected within approximately 10 ms of the onset of the electrical impulse. This indicates that the change-point determination method—threshold analysis of the local mean in the data—successfully extracted features relevant to the initial moments of myoelectric activation. The accuracy statistics, presented in Table 1, were calculated using a k-fold cross-validation approach with six folds (one per subject). This cross-validation process was repeated 1000 times on the entire dataset. Even without optimizing the distance metrics, weights, or computational costs, the classification certainty based on the change-point-derived features achieved a global average accuracy of 90.6% across all test cases. Further improvements were realized through Bayesian optimization. Specifically, a Gaussian process was used to explore a range of hyperparameter candidates. An acquisition function, observing the Expected Improvement (EI) of each hyperparameter set (as shown in Table 1), identified the optimal hyperparameter set for testing.

(1) $$EI\left(x,Q\right)=E_{Q}[max\left(0,\mu_{Q}\left(x_{best}-f\left(x\right))\right)\right]$$

where f(x) is the objective function, $x_{best}$ is the location of the lowest mean, and $\mu_{Q}\left(x_{best}\right)$ is the value of the lowest mean. This process was executed iteratively until a local minimum of the misclassification rate was reached. By stepping through 30 iterations of generational optimization, and applying different parameter combinations, Bayesian optimization yielded improved results as the classification error decreased with each iteration. As a result of the optimization, the accuracy of the trained model increased to 92.9% across all test cases, as shown in the confusion matrix in Figure 3, with a prediction speed of approximately 25,000 classifications per second. The highest-performing model used k=8 with a city-block distance metric and an inverse distance weight.

### 4.2. DNN Classification Results

**Figure 4:**
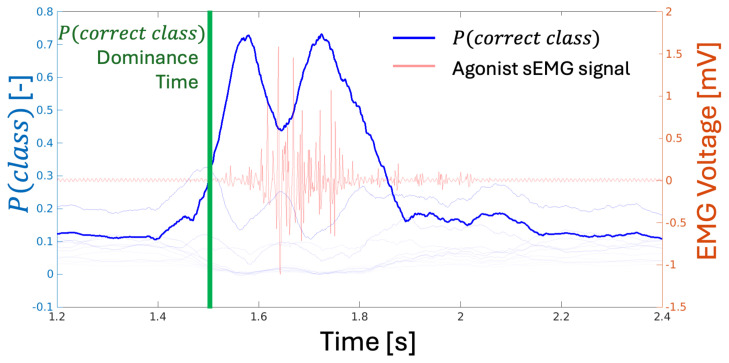
Plot showing an example of the evolution of P(class) over time during an EMG activation cycle. All 12 P(class) values are plotted, with each line’s opacity corresponding to its mean P(class). Notice the incline in the correct P(class) prior to the noticeable innervation of the primary agonist, which is the only EMG signal visible in the plot.

The neural network classification method demonstrated the ability to identify both the predicted class of each data point (as described in Section 3.3, where data points were rolling windows of variable length) within the time series and the associated probability distribution, P(class), for each prediction. The algorithm calculated the likelihood that each data point corresponds to a specific class. As illustrated in Figure 4, the probability of correct classification increases not only before the expected motion—accounting for the EMD delay of 50 ms to 200 ms following the initial electrical activation of the muscle group—but also before the sEMG signal indicates muscle activation of the primary agonist.

**Figure 5:**
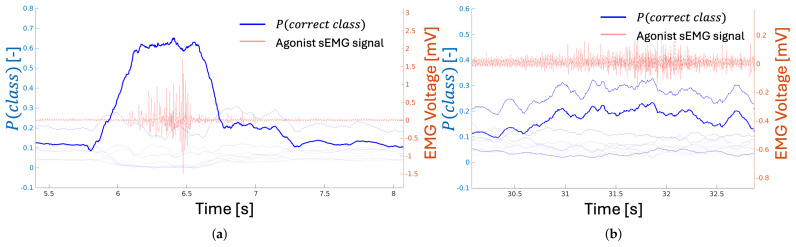
Examples of the trajectories that P(class) follows through trials of different magnitudes/motion groups. In subfigure (**a**), the prediction algorithm reaches certainty prior to the apparent myoelectric activation of the agonist muscle group, and that certainty remains dominant throughout the motion. This represents the majority of trials where the prediction algorithm performed nominally. However, in cases where no certainty was reached, the trajectories resembled those in subfigure (**b**), where there is no clear dominant prediction in terms of P(class), and, therefore, no reference point exists.

Since the determination of a class is based on the dominant P(class) within each rolling window of data, the first instance where P(correctclass) is highest can be considered the point at which the DNN classifier makes its most confident prediction. Using this definition of “confidence”, the classification network developed in this study was, on average, able to predict the onset of motion ahead of the EMG change point (as defined in Section 3.2) by an average of 10.2±32 ms across all trials. However, some results did not provide reliable classifications due to data peculiarities. For example, slow elbow extensions produced uncertain results because the network’s resulting P(correctclass) was insufficiently dominant to ensure certainty. Similarly, for fast arm extensions or slow wrist extensions, the rolling average window-based change-point detection method failed to identify adequate change points, leading to the absence of a reference point for predicting motion. These failure modes are illustrated in Figure 5.

## 5. Discussion

### 5.1. KNN Classification

There are two key aspects of the KNN classification results (Section 4.1) that warrant attention. The first is the ability to differentiate not only between types of motion but also between their magnitudes using information that is present prior to physical motion in the moments immediately after myoelectrical signal detection at the muscle (at the detected change-point onset). This result, in particular, suggests that it is possible to classify sEMG signals with corresponding motions prior to the initiation of physical motion: a true temporal “prediction” of motion, in line with the definition of prediction provided in this study (see Section 2.5 for the definition utilized). Additionally, while conventional wisdom has already acknowledged that an algorithm-discernible difference exists between the initial moments of different movement types [4], the ability to distinguish between different magnitudes of the same movement group has not been thoroughly investigated until now. The results of this study suggest that a machine learning model can detect differences between movement magnitudes a priori and that these differences are significant enough to achieve high classification certainty in nearly all cases, with only a few exceptions.

The second noteworthy finding from the KNN classification results is the determination of change points and their significance. We found that either very few parameters are required to achieve highly effective classification algorithms when predicting motion groups or that change-point analysis alone is sufficient for identifying the onset of myoelectric muscle activation for classification purposes, specifically for motions yet to occur. Recall that what this study refers to as “change points” were determined using a moving average cost-minimization analysis, as detailed in Section 3.2. This method is not novel; prior work has supported its use as a reliable means of determining relevant parameters in EMG analysis [17]. In the context of this study, the change points derived through this method were shown to be both relatively immediate and reliable in detecting muscle activation at the onset of a given muscle group’s activation.

### 5.2. DNN Classification

In a field dominated by post-hoc EMG gesture classification work, where prediction is rarely addressed, the results from the neural network classifier experiment in this study present several novel findings. First, much like the KNN-based prediction in Section 5.1, the neural network classification method was able to “predict” motion. The certainty of classification for any particular motion became dominant prior to the commonly acknowledged EMD closing. Furthermore, the point of greatest certainty, where P(correctclass) becomes dominant, was reached even before the algorithm-calculated change point and the visual determination of activation. The majority of trials resulted in correct predictions of motion type and magnitude ahead of the apparent electrical activation of the agonist muscles for those motions. This result was repeatable; as shown, all but a few trials demonstrated that the probability for the correct class led to the algorithm-calculated change point, which, in turn, preceded the physical motion. This finding is consistent with the existing literature suggesting that muscle activation leads motion by some amount of time.

This phenomenon points to the possibility that there are features in sEMG behavior that precede the activation of a muscle group. While the suggestion of such features is not new, as many prior studies [8,9,10] have investigated what is often referred to as “readiness potential”, these prior works support the hypothesis that detectable and comprehensible myoelectric behaviors indicate an impending activation cascade and, thus, muscle group activation and motion. Although this hypothesis has not been robustly supported—particularly the claim that this pre-activation behavior is repeatable, intelligible, or parametric—the results from the DNN portion of this study strongly suggest that there may be at least a non-parametric, pattern-driven relationship, if not a more complex parametric feature.

The exact biological cause of such a preemptive marker feature, if it exists, is beyond the scope of this study and is left for future work. However, the observation of relatively consistent prediction behavior, even before the detection of activation potential, warrants further investigation. Future experiments could aim to isolate and investigate the cause of this behavior and the features on which these predictions depend.

### 5.3. Limitations

Below are some limitations that, when addressed in future studies on this matter, may provide value. They are organized into categories of improvement.

#### 5.3.1. Algorithm Performance and Validation

There are certain limitations in both the KNN and DNN classification studies presented in this work. The primary limitation is the low number of subjects. Such machine learning-based approaches generally require large amounts of training data, which was satisfied even with the limited number of subjects in this study, as each subject produced a sufficient amount of data. However, a lack of generalizability is demonstrated when using smaller subject numbers; therefore, this study is not sufficient to make universal claims. Of particular note on this matter are the results of the DNN inference attempt on the slow elbow extension action, in which the predictions were inconclusive; it is possible that due to a lower-than-necessary data point count, there was no “consensus” reached in the training process. Future studies with similar methods should consider recruiting more or a wider variability of subjects to counteract this potential shortcoming. Furthermore, it is possible that, due to optimization processes and network design, these prediction algorithms were overfitted to the given subjects and thus would yield significantly different results in an ablation analysis involving new subjects. Although this study does attempt to mitigate this possibility through a k-fold cross-validation analysis, the presence of 100% classification confidence (as seen in Figure 3) may indicate either a particularly distinct feature set or signs of overfitting. This should be addressed in future studies through either much larger subject pools or ablation analysis with varying chunk sizes.

Additionally, while the existence of EMD is by no means a poor assumption to make based on relatively robust prior work, explicitly designing experiments to observe EMD as a means of confirming ground truth in future studies would make subsequent results more independently verifiable; it can feasibly be argued at the moment that EMD is a motion-specific phenomenon and must be observed in the particular context of the parameters of the study presented here. Such an experiment design would involve, for example, capturing the delay between myoelectric activation and physical motion through kinematic measurements such as goniometers, motion capture, or similar sensors.

#### 5.3.2. Real-Time Practicality

Furthermore, there are limitations to the practicality of the particular method presented in this study. Due to the filtering and change-point identification methods used, the entire dataset must be available prior to inference, which limits its real-time deployability. Although this study in isolation does not make claims to real-time inference capabilities, and while we suggest that the results in this study indicate the possibility of a similar prediction capability in a real-time-oriented experiment using the same physiological phenomena, such a study must be executed to confirm that the capability demonstrated in this study is achievable with different filtering and feature selection methods. An additional consideration is the computational cost of the processes involved in the prediction, even when discounting steps that require the entire dataset to be available prior to inference. For example, the DNN requires a non-zero amount of time to execute an inference, and this time scales with the complexity of the network, which, in turn, often affects the accuracy of the prediction. Therefore, computational hardware cannot be ignored. In this study, no significant consideration was given to this computational resource limitation or the consequences of network complexity, both of which are certainly factors when considering real-time applications. However, in future studies that aim to robustly establish real-time viability, these factors should be thoroughly investigated, especially when considering mobile form factors.

#### 5.3.3. Study Assumptions

Finally, this study sets assumptions (as outlined in Section 2.5) that can present areas of improvement, specifically regarding the selection of muscles and their significance in the selected motion groups examined in this study. It is worth highlighting that while the muscles of this study were selected mostly based on size and ease of sensing, not all motions utilize all muscles, and at times, none of the muscles may be significantly recruited in the prescribed motion (i.e., wrist motions, which are usually dominated by activation of the family of Extensor Carpi (EC) muscles adjacent to the Brachioradialis (BR) observed in this study). Adding to this limitation is the fact that probes were self-placed under supervision, as opposed to being placed by experiment staff, thus introducing additional variability in data quality. It is possible that, for the wrist motions in particular, results were skewed by the fact that it is likely the BR channel was seeing cross-talk from these EC groups nearby. While in this study, this auxiliary signal (or secondary signal from primary recruitment nearby) appears to be sufficient to demonstrate accurate prediction of wrist motions, it should certainly be confirmed through direct observation using additional sEMG probes in future studies. Consistent with the assumptions made, it is a natural extension to investigate similar motions with additional sensors on additional muscles, providing more channels of information for the prediction algorithms. Furthermore, the range of selected motions was limited. While the motions selected were evidently sufficient for the demonstration of the concept, future studies should incorporate more varied motions that recruit a wider range of muscles. In particular, co-contracting motions should be included in future studies, as they represent often large muscle recruitment but result in little to no kinematic motion and instead change joint stiffness. Thus, there are two aspects that should be specifically probed in future studies: (1) the classification of more motions, including co-contracting motions and their effect on prediction classification performance, and (2) the performance of the overall methodology presented in this study, which should be tested with more channels of sEMG data to determine whether more numerous/accurate sensor readings do, in fact, improve prediction quality and accuracy.

### 5.4. Potential Applications

This study demonstrates two ways in which relatively low-complexity learning algorithms can be used to predict motion by generating a classification of human subject motion prior to the initiation of physical motion with a time difference on the order of tens to hundreds of milliseconds. There are potential applications of this development both in its classification form demonstrated here and in a much larger range of utilization with further development of the paradigm.

We propose that there are applications even for the proof-of-concept level of performance shown in this study. For example, such a classification of motion ahead of physical motion can be utilized in mitigating adverse physiological actuation, such as seizures. A hypothetical classification network trained to identify impending seizures hundreds of milliseconds in advance may be used to mitigate accidents during equipment or vehicle operation. Furthermore, it can be used to streamline gesture-based interfacing, such as in VR applications.

The more interesting prospect, based on the developments shared in this study, is the possible applications that further development will enable—most importantly, the utilization of a regression algorithm for a similar purpose. The ability to predict the trajectory of the human agent with greater granularity has massive potential in the human–robot interaction (HRI) field. If a reliable regression-based prediction of a limb’s trajectory prior to motion, for example, can be achieved, it would advance many tandem HRI teams (such as powered augmenting exoskeletons) beyond the state of the art, as discussed in Section 2.2. We hypothesize that such a system would have dramatically increased fluency, trust, and energetic efficiency.

## 6. Conclusions

In this study, we described the reasoning, hypotheses, methods, and results of two experiments focused on the design and evaluation of motion prediction classification algorithms. These algorithms were based on a KNN approach and a DNN approach, both assuming the existence of EMG features that precede noticeable muscle group activation and indicate the impending onset of motion. The KNN approach leveraged this hypothesized phenomenon for classification. In this approach, data from subjects performing various motions, differing in type, speed, and size, were pre-processed for features at points determined by a change-point algorithm. This algorithm identified the time at which myoelectric activation begins, and the resulting features were used to train a classification model. The KNN model achieved a classification accuracy of 92.9±2.9% for both motion type and speed based on features collected around the change-point moments, which are presumed to precede the physical motion of the corresponding muscles by tens to hundreds of milliseconds.

In a second experiment, a DNN-based approach was used to predict the motion group based on rolling windows of raw sEMG data. The neural network produced a probability distribution of all available classes for each window, providing a prediction for any given point in the time series. Using the same raw data as the KNN approach for training, the DNN classification algorithm was able to not only produce high classification probabilities prior to motion, accounting for electromyographic delay (EMD), but also generate high-probability predictions ahead of sEMG change points with a lead time of 10.2±32 ms. In other words, the DNN algorithm successfully predicted motion before the visually identifiable onset of myoelectric muscle activation in the vast majority of test cases.

## Figures and Tables

**Table 1:** KNN classification prediction performance.

Optimization	Accuracy	σ	k	Distance Metric
Optimized	92.9%	2.9%	8	City-block
Unoptmizied	90.6%	2.8%	10	Euclidean

**Table 2:** Performance breakdown of the DNN prediction method for each motion.

Label			Mean lead time [s]		σ	CI 95%
Fast	Arm	Flexion	0.208		0.081	0.027
		Extension	Change point inconclusive			
		Abduction	0.158		0.080	0.026
	Elbow	Flexion	0.020		0.072	0.024
		Extension	0.074		0.047	0.016
	Wrist	Extension	0.067		0.168	0.057
Slow	Arm	Flexion	0.143		0.082	0.027
		Extension	0.155		0.274	0.093
		Abduction	0.322		0.245	0.083
	Elbow	Flexion	0.273		0.179	0.061
		Extension	Prediction certainty not reached			
	Wrist	Extension	Change point inconclusive			

## Data Availability

The datasets presented in this article are not readily available because the data are part of an ongoing study. Requests to access the datasets should be directed to Bon H. Koo (bkoo1104@mit.edu) and Lonnie G. Petersen (lgpeters@mit.edu).

## Abbreviations

The following abbreviations are used in this manuscript:

EMD	Electromechanical Delay
KNN	K-Nearest Neighbor
DNN	Deep Neural Network
EMG	Electromyography
DoF	Degrees of Freedom
TPR	True Positive Rate
FNR	False Negative Rate
DAQ	Data Acquisition
HRI	Human–Robot Interfacing/Interaction
